# A Contribution to the Study of the Microbe of Rabbit Septicæma

**Published:** 1887-01

**Authors:** Theobald Smith

**Affiliations:** Bureau of Animal Industry, U. S. Dept. of Agriculture


### ART. III.—A CONTRIBUTION TO THE STUDY OF THE MICROBE OF RABBIT SEPTICÆMIA.

BY THEOBALD SMITH, M. D.
*Bureau of Animal Industry, U. S. Dept. of Agriculture.*

The accidental discovery of this microbe in two rabbits was the starting point of some experiments with a disease which has already received considerable attention at the hands of Davaine, Koch, Gaffky and others. The following rather incomplete observations confirm the results of the above mentioned observers as to the existence of a specific micro-organism of septicæmia in rabbits, and also call attention to some marked differences in its effects upon animals, indicative of a difference in virulence between what we might call provisionally the European and the American variety. In the following pages the source of the microbe, its microscopic appearance and mode of growth in various culture media are first described; after which is given an account of the inoculation experiments, and finally a comparison between the results obtained and those of former observers.

A lot of ten rabbits purchased for experimental purposes died soon after their arrival, so that in a few weeks only two were left. The animals had been sent by express over a hundred miles from the country. The earlier deaths were, quite naturally, referred to the ill effects of the journey, and stress of work prevented any careful investigation. Two were examined, however, and in one of these, which we shall denominate No. 5, the only lesions were a fibrinous exudate on the left pulmonary pleura and considerable congestion of the kidneys. Cover-glass preparations of the spleen revealed the presence of a large number of oval cocci corresponding very closely, in appearance, to the bacterium of septicæmia in rabbits as described by Gaffky.* In the liver the same forms, in general, slightly larger, were present in large numbers.†

---

*Mittheilungen a. d. Kais. Gesundheits amte (1881), I.

†In this connection it might be well to make a few suggestions which have frequently come to the writer in examining cover-glass preparations. The bacteria present in an internal organ, such as the spleen for example, will, as a rule, multiply after death if the surrounding temperature be favorable. This multiplication, we must bear in mind, takes place under changed conditions. The resistance offered by the vital activity of the cells is gone, to be sure, but, on the other hand, the blood and lymph currents are checked by death, which thus limits the food supply. Respiration is interrupted, which forces the bacteria to live virtually without oxygen. To illustrate this fact, we find now and then an active growth of the bacillus of malignant œdema (the spores of which are usually present in the alimentary canal) in internal organs. This growth is probably post-mortem and induced by the absence of oxygen. Nutrient liquids inoculated from such organs always remain sterile. The bacteria multiplying in the tissues of dead animals may under these changed conditions vary somewhat in size. This may serve to explain the larger size of the bacteria in the liver of No. 5. It might be said that the same

A second rabbit (No. 6) had suffered from peritonitis as manifested by the roughened condition of the peritoneum and the adhesion of the coils of the large intestine to one another and to the bladder. The lungs were considerably reddened, the epicardium covered by a whitish exudate which, on microscopic examination, was found to consist almost entirely of oval bacteria. The majority of these were much smaller than those found in No. 5, as if in a state of active multiplication. Now and then an individual could be seen stained precisely like those in No. 5. From this exudate a liquid culture was inoculated in which an immotile oval microbe developed which could not be distinguished from the microbe found in cultures from No. 5, to be presently described. On gelatine plates the colonies of both were identical in appearance. Only two mice were inoculated from this culture, each receiving $\frac{1}{8}$ cc. hypodermically. One died on the second day, and in the liver and the deeply congested spleen the oval microbes were present in moderate numbers. No other experiments were made with the germ from No. 6, all the rest being made with cultures derived from No. 5. There is no reason, however, why the microbes from these two sources should not be considered the same.

*General characters.*—The microbe found in the spleen of rabbit No. 5, and in the animals which died from the effects of inoculation with pure cultures, to be subsequently described, is best studied in cover-glass preparations. A thin film of spleen

---

conditions prevailed in both spleen and liver of this animal, but this is only partially true. The metabolic activity of one organ produces substances quite different from those in the other, and the presence of these substances may also affect the growth of the microbes. It is a common observation to note in different liquid and solid culture media slight changes in size and form. So-called involution forms may arise which are characterized by larger size and abnormal shape. Division is retarded and chains of individuals appear often very imperfectly divided. Such observations may be said to represent the aggregate experience of impressions not easily demonstrated to others, but nevertheless convincing to the observer. They indicate the importance of relying, within certain limits, upon dimension and form only when the conditions are precisely alike. Slight variability in the size of the same species of bacteria, according to the culture media employed, is just now beginning to receive some credence, and it emphasizes the importance of testing a given species in all possible media and upon animals in order to obtain definite means of diagnosis. *See Frankel and Simmonds: Die ætiologische Bedeutung d. Typhus-bacillus.* 1886.

pulp is rubbed upon a cover-glass. After the usual manipulations of heating, etc., this is stained in an aqueous solution of methyl violet for 3 to 5 minutes, and after washing and drying, mounted in xylol balsam. Examined with a one-eighteenth homog. objective, the microbe appears as an oval body, which is deeply stained at the two poles of the longer axis, while the central portion is nearly transparent. The stained portions have the form of segments of a circle or crescents; the unstained central portion includes from $\frac{1}{3}$ to $\frac{1}{2}$ the optical area of the entire oval. In such preparations the dimensions of the average form are 1.2 $\mu$ in length and 8 $\mu$ in width. Longer ovals are sometimes observed measuring 1.6$\mu$. In these, the increase in length corresponds to an increase of the unstained area. Shorter forms, 1 u by .6 u are now and then seen. When cultures in liquid media are dried and stained as described, many nearly spherical forms are seen which are smaller and which stain more or less uniformly. If, however, attention be directed to the edge of the dried film, where a large number of bacteria are usually gathered together, many will be found closely resembling those in the blood and tissues as regards size and mode of staining; the smaller forms may result from a rapid multiplication.

The presence of the microbe in the internal organs may be easily demonstrated in sections of tissues hardened in absolute alcohol. They appear very well when the sections are stained over night in aniline water methyl violet and subsequently treated with $\frac{1}{2}$ % acetic acid, alcohol and turpentine, and mounted in xylol balsam. They appear as a rule somewhat smaller than in cover-glass preparations and do not show the polar stain very clearly. In sections of the spleen they are found in groups in the pulp, but not in the malpighian corpuscles. In the kidney they seem to be limited to the larger blood vessels, where they lie scattered among the blood corpuscles. In the liver they occupy the capillaries of the acini in extensive groups. By carefully focussing, these groups are resolved into flat layers attached to the walls of the vessels, but never into plugs clogging them.

If a tube containing some nutrient liquid, such as a neutralized infusion of beef containing one per cent. peptone, be inoculated with blood or other fluid containing this microbe, a faint opalescence is manifested within 24 hours, which is more easily seen on shaking the tube. After a few days a very thin, translucent pellicle appears on the surface of the liquid, gradually thickening into an irregular whitish layer from ½ to 1 mm. thick. If not disturbed this membrane will remain on the surface for weeks. A deposit, on the bottom of the tube, from fragments of membrane and suspended matter, gradually accumulates while the culture liquid once more clears up.* If a drop from such a culture be examined at different stages, in a cell under the microscope, the oval microbes exhibit Brownian movement only. They are therefore *not motile.*

On gelatine plates the centers of growth become visible to the naked eye within 24 to 48 hours, according to the temperature of the room. They appear as minute, spherical sharply outlined masses with homogeneous disc, which is very pale by transmitted light. They do *not liquefy gelatine.* On the surface of the gelatine, they appear as flattish, irregular patches with very thin border. If a tube culture, in a slightly alkaline beef infusion peptone gelatine, be made, by piercing the gelatine with a platinum wire previously passed into the parenchyma of the liver or spleen or dipped into blood, a number of isolated spherical masses will appear in the needle track, within two days. These if numerous may coalesce into a whitish line. The surface growth is quite vigorous. Milk is not altered in appearance after inoculation. Blood serum from the cow does not appear to promote growth better than the nutrient gelatine. On potato no multiplication takes place. The writer has occasionally resorted to a method of stimulating the growth upon potato, of bacteria, which ordinarily refuse it is a pabulum, by saturating the cut surface with some sterile nutritive liquid. This for want of time was not tested for the microbe under consideration. In bulbs containing culture liquid from which the air has been more or less completely exhausted, no clouding takes place. The germ cannot be considered anærobic therefore. Culture tubes inoculated and then exposed to

*The use of liquid cultures first introduced more systematically by Pasteur, has been almost superseded by the use of solid media in Germany. (See *Med. News*, 1886, Nov. 20).

a temperature of 58° C in a water bath, for 15 minutes remain sterile, those exposed for 10 minutes, become turbid. Tubes inoculated from cultures two to three weeks old, are apt to remain sterile indicating that the microbe does not retain its vitality very long. This fact, together with the low thermal death point, demonstrates the absence of any resistant spore state.

*Inoculation experiments.*—A gelatine tube culture inoculated by means of a platinum wire from the liver of rabbit No. 5, remained sterile.* A tube of beef infusion with peptone inoculated with a loop of blood from the heart became faintly clouded within 24 hours and contained the immotile ovals only. Two mice inoculated by a subcutaneous injection of a few drops, died, one on the following day the other on the second day. In the spleen and liver of the latter the injected bacteria were present in large numbers, but were absent in the former. A liquid culture from the heart of this mouse contained the injected microbe only.

On June 9th, 1886, a tube of beef infusion peptone was inoculated from a colony on a gelatine plate prepared from the original liquid culture from No. 5. Next day the faintly clouded tube was found pure. Not knowing as yet the nature of this germ, two rabbits (No. 10, 11,) were inoculated by giving them a rather large dose of this culture, ¼ cc. Both rabbits were found dead on the morning of June 12th, within 48 hours after inoculation At the point of injection, a very thin whitish, pasty looking deposit was found in the fascia covering the muscles, extending over an area of 3 or 4 sq. cm. and consisting of leucocytes and an immense number of the oval microbes. The spleen was gorged with dark blood and friable, the lungs reddened. On the rectum beneath the serosa, and in the meso-rectum were numerous ecchymoses; beneath the corresponding mucosa punctiform extravasations. The stomach was filled with food which was covered with a layer of tenacious mucus. The spleen and liver were crowded with the specific microbe. The lesions in No. 11, closely resembled those just given. There was some serum in the peri-

*This preparation of nutrient gelatine was found, later on, not sufficiently alkaline for this microbe.

toneal cavity and the lesions in the rectum more pronounced, the extravasations beneath the mucosa assuming the form of small bulging hæmatomata. The specific microbe was found in cover-glass preparations of blood from the heart, of the liver, spleen, lung and kidney. Liquid cultures from the blood (heart) and cultures in tubes of gelatine from the liver of both animals contained the microbe of septicæmia as described, all pure. Two pigeons were inoculated subcutaneously, one near the keel, the other in the shoulder, with ¼ cc. of the third liquid culture, 24 hours old, of blood from rabbit 11. One died seven days later during the writers' absence and was not examined, the other remained well for weeks after. A guinea pig inoculated subcutaneously with ¼ cc. and two pigs with 1 cc. each of the same culture were not affected.

To determine more definitely the pathogenic effect of this microbe, it became necessary to continue the inoculation experiments. A liquid culture inoculated from the gelatine tube culture of rabbit No. 10 (liver), was faintly clouded in 48 hours. With it the following inoculations were made hypodermically. Two rabbits (No. 12, 13), two guinea pigs and a young rat received ¼ cc. each, two fowls 1.5 cc. each. Of these animals, the rabbits died within 24 and 48 hours respectively. One of the guinea pigs died in 5 days. The others were well three weeks later. In rabbit No. 12, there were about half a dozen small ecchymoses in the connection tissue at the point of inoculation—nothing else. The rapidity with which the virus had acted, left the organs microscopically unchanged, if we except a reddening of the cortex of the kidney. The oval microbe was revealed by its peculiar stain in preparations of the spleen, liver, blood from the heart and kidneys, being least numerous in the last mentioned organ. In No. 13, a whitish thin deposit (pus), had formed in the connective tissue at the point of inoculation precisely like that observed in Nos. 10, 11. The spleen was enlarged, dark, friable, the serosa of part of the small intestine reddened. The specific microbe was present in the spleen, liver, kidneys and blood from the heart in considerable numbers. From the blood pure liquid cultures were obtained. Gelatine tubes inoculated from the spleen and liver of each animal remained sterile. The cause was found to be in an insufficient alkalinity of the medium, as the microbes grew vigorously in another more alkaline preparation of gelatine inoculated from the blood cultures. In the guinea-pig there was slight engorgement of the blood vessels, at the point of inoculation. Severe peritonitis was indicated by an inflammation of the serous covering of the abdominal walls, the intestine and bladder and some effusion. The spleen was enlarged, dark, very soft; the liver almost pulpy and covered with a gelatinous exudate, about ½ mm. thick, readily peeled off. Lungs œdematous and congested; a few subpleural ecchymoses present. Bacteria were few in number in the internal organs. Two liquid cultures of the blood were found pure next day.

These results were confirmed by another series of inoculations, in which the microbes were introduced beneath the skin on a platinum loop directly from gelatine tube culture (Nos. 10, 11, liver). The skin having been incised, the zooglœar mass was introduced into the small pouch thus made. In this way two mice were inoculated from rabbit No. 10; from No. 11, one rabbit and three pigeons. One mouse died three days later. No microbes in spleen or liver. The other mouse and the pigeons remained permanently well. The rabbit (No. 14) showed some lameness, though continuing to eat until it was found dead one week after inoculation. The lesions in this animal were different from those of the preceding cases. The seat of inoculation on the thigh was occupied by an extensive patch of whitish pasty infiltration, which was continued as two thick cords along the median portion of the abdomen as far as the sternum; the lateral aspect of the abdominal wall, on the inoculated side was likewise infiltrated with pus, which contained numerous oval microbes staining very feebly. The abdominal cavity was invaded by an intense plastic peritonitis, matting all the viscera together with a blood-stained gelatinous exudate. The large intestine was covered with patches of extravasation. The specific microbe was present in very small numbers in the various organs. Two liquid cultures from the heart were pure, none others were made. This peculiar case will be discussed later.

The results thus far pointed to a comparative insusceptibility of all but the rabbits; these seemed to possess no power of resistance. A final trial was made to confirm these results. A liquid culture, one day old, prepared from a gelatine tube culture of rabbit 13, faintly turbid, and containing the characteristic microbe only, was used. Three mice received each $\frac{1}{8}$ cc., one young guinea pig $\frac{1}{4}$ cc., two fowls and three pigeons 1 cc. each, two rabbits $\frac{1}{40}$ and $\frac{1}{8}$ cc., respectively. Of these animals the fowls and guinea pig remained unaffected. The three mice were sick for some days but regained their wonted activity. The two rabbits died within 48 hours after inoculation. Two out of three pigeons died in four and six days respectively. In rabbit No. 16 (which had received but $\frac{1}{40}$ cc.) there was considerable local reaction; the muscular tissue over an area of a sq. cm. was softened; over the muscles of the thigh, on the inner aspect, there was a thin pasty layer of pus, with scattered points and patches of extravasation. The suppurative change extended towards the dorsum in a thick cord-like mass, involving a portion of the lateral wall of the abdomen, which was whitish and softened. Beginning peritonitis manifested by delicate strings of exudate across the intestines. Liver pale, spleen enlarged, dark and soft, cortex of kidney injected. Bacteria comparatively few in spleen and liver, quite numerous in blood from the heart. A liquid culture from the heart and a gelatine tube culture from the liver were both pure. In rabbit No. 17, which had received at least five times the quantity given to No. 16, the local reaction was limited to a slight infiltration of the fascia covering the muscles. The gall-bladder was distended and the surrounding tissues stained with bile. Other changes like those above described. Both spleen and liver contain numerous microbes, the heart but few. Successful cultures made from blood and liver.

In one of the pigeons (No. 3) which had received 1 cc. beneath the skin and died in four days, the muscular tissue was slightly necrosed superficially, forming a brownish-red sequestrum. The specific microbe was abundant in spleen and liver. A liquid culture from the heart and a gelatine tube culture from the spleen contained this microbe only.

The other pigeon had been inoculated by pushing the needle into the muscular tissue of the pectoral making it an intramuscular injection. The necrosis in this case was more extensive and deeper. On section it was more or less honeycombed, including small masses of normal muscular fiber. No bacteria was found in the internal organs and a liquid culture from the heart remained sterile.

The disease was thus transmitted, by means of pure cultures of varying age, from rabbit No. 5 to Nos. 10 and 11; from No. 10 to Nos. 12 and 13; from No. 11 to No. 14, and from No. 13 to Nos. 16 and 17. The inoculated rabbits invariably succumbed, nearly all within 48 hours. Opposed to these very positive results we have chiefly negative ones to record with reference to other animals. Three out of four guinea-pigs and 4 fowls proved insusceptible, and but 3 out of 7 pigeons died. Even mice require a large dose. It is very probable that none of these animals would have been killed by the minimum dose needed to kill a rabbit, when we consider the comparatively large quantities with which they were inoculated. We must therefore regard the disease as peculiar to rabbits, unless we are dealing with an attenuated variety.

The experiments are too few in number to warrant any extensive generalizations, but it may be well to suggest a few lines of thought which may lead to further investigation. In glancing over the autopsy notes, we find that the local reaction grew in intensity with the duration of the disease, that the smaller dose produced the severer reaction, that cultures not suspended in liquids (*i. e.*, in masses from gelatine culture) act like small doses of liquid cultures; and finally that a very slight local reaction is associated with an abundance of bacteria in the blood of the internal organs. For these rather strange phenomena we would offer the following explanation: When introduced beneath the skin the bacteria may become disseminated and multiply in the lymph or blood channels. Those that enter the blood meet with a greater resistance than those in the lymph channels, hence, when a small quantity is injected, those that enter the blood vascular system are either destroyed or are placed in conditions in which multiplication is prevented. Meanwhile some follow the lymphatics, multiply, induce suppuration and inflammation of the large serous cavities. When large quantities are injected the resistance of the blood is overcome at the outset, multiplication goes on rapidly, and after death the bacteria are found in abundance in all the organs, though a local reaction may be absent. In the former case they are few in number, although the disease may be prolonged with extensive local lesions.

When the bacteria are introduced as they grow upon solid media, in a mass, we obtain the effect of a small liquid injection (rabbit No. 14), probably because they are not in a condition to be rapidly carried into the circulation. The bacteria creep along the lymphatics, invade the serous cavities and finally produce death. In this case also the disease is prolonged, but the organs contain few bacteria after death.* An injury to a blood vessel at the time of inoculation might materially change the anticipated results.

This microbe was first studied by Koch,† who produced the disease by the subcutaneous injection of putrid meat infusion. At the same time, Koch describes another disease produced by injecting putrid substances—pyæmia in rabbits—in which the local lesions and extensive peritonitis remind us of a few of the cases described in the preceding pages. This disease was caused by small micrococci (.25 $\mu$), while in the writer's experiments pure culture of the microbe of septicæmia were obtained from these same cases. Somewhat later the same disease was studied by Gaffky.* Rabbits and mice invariably

* The fact previously mentioned that bacteria are apt to multiply *post mortem* must not be forgotten in this connection. Experimental animals die most commonly at night. When the temperature is very high, as during the summer months, there may be an appreciable increase in the number of bateria before an examination can be made. This important fact has been forced upon the writer's attention in other investigations and has been noted by Frankel and Simmonds in typhoid fever (*loc. cit.*) These investigators made use of the *post mortem* multiplication of the specific bacillus of this disease in studying the disposition of bacillar groups in the spleen. This organ, removed *in toto*, was wrapped up in a cloth saturated with a solution of mercuric chloride and kept in a warm room for a certain length of time, usually for one or two days. It must also be borne in mind that bacteria multiply *in situ* after death. The cessation of all circulation will prevent their distribution. Hence an organ comparatively free from bacteria at the time of death would remain so, while one in which they are widely distributed would contain large numbers after a time, there being many centers of multiplication.

† Wund infectiouskrankheiten S. 59.

succumbed to inoculation in from 16 to 20 hours. There was no local reaction. Its absence might be explained by the brief duration of the disease, for, in the cases recorded in the preceding pages, the severity of the local lesion was proportional to the duration of the disease, there being none whatever in one (rabbit No. 12), which died within 24 hours after inoculation. In producing the disease originally by the injection of contaminated water, Gaffky found a case of peritonitis, which he regarded as due to another microbe, although the microbe of septicæmia was present in the blood. In view of the above experiments, we should consider the peritonitis as belonging to the disease, more especially as it lasted over three days. Gaffky found fowls pigeons, and sparrows susceptible, but guinea-pigs and white rats insusceptible. The microbe remained virulent for months in artificial cultures.

Dr. Cheyne, in some recent experiments on the conditions of infection,† states that the experiments of Koch and Gaffky with diluted blood from septicæmic rabbits, leave no doubt that even a single microbe is sufficient to kill a rabbit. He himself endeavored to determine the number necessary to destroy guinea-pigs. The injection of small doses proved fatal to nearly all. A peritonitis developed in some cases, in others the microbe seemed to be most abundant in the blood. Gaffky found guinea-pigs insusceptible. The discrepancy may be due to the size of the dose and the mode of inoculation.

The microbe found by Dr. Sternberg in his own saliva, which produces septicæmia in rabbits,* differs both morphologically and in its pathogenic effect from the microbe under consideration.

Taking into consideration all the facts concerning this microbe‡ which have been brought to light by these observers,

* Mittheilunger a. d. Kais. Gesundheits arute I, S 93.

† *British Medical Journal.* 1886–ii., p. 203.

‡ This organism illustrates very well the change in terminology which has been going on in the field of bacteriology. It was first described by Koch as a micrococcus; Gaffky called it a bacterium and in the recently published tables of Eisenberg (*loc. cit.*) which were compiled under Koch's supervision, it is called a bacillus. The original name of micrococcus seems most reasonable.

* Am. Jour. of Med. Sciences, 1885, p. 106; 1886, p. 123.

we must adopt one of two views. The microbes under consideration may be entirely different specifically, or else the one found by the writer may be an attenuated form which has lost the power of multiplying in all animals but the rabbits. This is a more plausible assumption than one which would credit American pigeons, fowls, etc., with a greater power of resistance. Though some of the pigeons inoculated by the writer died, they cannot be regarded as really susceptible organisms, a susceptible animal should succumb to a minute dose, and these received large ones. A micro-organism which has marked pathogenic properties with reference to one species must necessarily be injurious to other animals, if only in a slight degree. When introduced into the system in large numbers, it may be able to reduce suddenly the vitality of the animal sufficiently to gain permanent foothold and finally destroy it. The same reasoning might be extended to the mice and the guinea-pig. This argument, however, does not invalidate the use of large quantities of virus in studying its effects upon comparatively insusceptible animals.

It has been asserted that the microbe of rabbit septicæmia and of fowl cholera are probably identical. This assertion, based upon that of German investigators, cannot be sustained with reference to the microbe under consideration. Moreover, a recent observer, who, without doubt, had the real virus of fowl cholera, describes the microbes as spherical cocci .3 to .5 $\mu$. in diameter.* There may be two forms in Germany to which fowls are susceptible, one identical with the microbe of rabbit septicæmia, the other a true micrococcus. Whether both are capable of causing epidemics, or whether one only is the true virus of fowl cholera, as observed by these investigators, can not be answered on this side of the Atlantic.

The question as to the distribution in nature of this microbe is of considerable interest, involving, as it does, the more important one of the distribution of all the so-called ubiquitous pathogenic micro-organisms, which produce suppuration, septicæmia and pyæmia in man. Assuming that the microbe first described by Koch, and studied more min-

*Deutsche Ztschr. *f.* Thiermedicin u. vergl. Pathologie. Supplement, 1885.

utely by Gaffky, is identical specifically with the one now under consideration, but one of them modified by conditions not at present determinable, it may have a wide distribution and yet be very rare. Koch preduccd it twice by injecting putrid meat infusion. How often putrid substances were injected into rabbits before these two successful cases were obtained, is not stated. Gaffky remarks, however, that injections were made by Koch upon a large number of animals before this specific form of septicæmia was produced. The same difficulty was experienced later by Gaffky, who succeeded at first with water from a contaminated stream in Berlin (Panke), but as the weather grew cold this source of the microbe also failed. In this connection the question arises, How were the two animals infected which furnished the writer with the original cultures? They are the only cases of spontaneous origin on record, as they had all been obtained from the country in a healthy condition. We may assume that the microbe lives and retains its virulence outside of the animal organism like the virus of anthrax, and gains entrance through some lesion of the skin. Unlike the bacillus of anthrax, its feeble growth in artificial media stamps it as no very hardy germ.

There are many other problems concerning this germ left unsolved, such as the effect of feeding, of inoculation upon animals with the septicæmic blood itself to determine any possible attenuation in cultures with reference to the animals not found susceptible, its resistence to various mechanical and chemical agencies, etc. The experiments and observations recorded seem to demonstrate fairly well the following points:

1. There is a microbe distinguishable by definite morphological and biological characters which is invariably fatal to rabbits, but which has only a feeble pathogenic power with reference to guinea-pigs, mice, pigeons, and fowls.

2. The kind of lesions consequent upon subcutaneous inoculation in rabbits and their severity depend upon the number of microbes introduced and upon the mode of inoculation.

September, 1886.

